# Effects on Lignin Structure of Coumarate 3-Hydroxylase Downregulation in Poplar

**DOI:** 10.1007/s12155-012-9218-y

**Published:** 2012-05-24

**Authors:** John Ralph, Takuya Akiyama, Heather D. Coleman, Shawn D. Mansfield

**Affiliations:** 1 Department of Biochemistry, Enzyme Institute, University of Wisconsin-Madison, 1710 University Avenue, Madison, WI 53726 USA; 2 Department of Biological Systems Engineering, University of Wisconsin-Madison, 460 Henry Mall, Madison, WI 53706 USA; 3 DOE Great Lakes Bioenergy Research Center, and Wisconsin Bioenergy Initiative, University of Wisconsin-Madison, Madison, WI 53706 USA; 4 Wood Chemistry Laboratory, Department of Biomaterial Sciences, the University of Tokyo, Bunkyo-ku Tokyo, 113-8657 Japan; 5 Department of Biology, Syracuse University, 460 Life Sciences Complex, 107 College Place, Syracuse, NY 13244 USA; 6 Department of Wood Science, University of British Columbia, Vancouver, BC V6T 1Z4 Canada

**Keywords:** Gene downregulation, NMR, Thioacidolysis, Digestibility, Biomass conversion, Lignin composition

## Abstract

The lignin structural ramifications of coumarate 3-hydroxylase (C3H) downregulation have not been addressed in hardwoods. Such information is required to accompany an assessment of the digestibility and bioenergy performance characteristics of poplar, in particular. Structurally rich 2D NMR methods were applied to the entire lignin fraction to delineate lignin *p*-hydroxyphenyl:guaiacyl:syringyl (H:G:S) levels and linkage distribution changes (and to compare with traditional degradative analyses). C3H downregulation reduced lignin levels by half and markedly increased the proportion of H units relative to the normally dominant G and S units. Relative stem H unit levels were up by ∼ 100-fold to ∼ 31 %, almost totally at the expense of G units; differences in the lignin interunit linkage distributions were more subtle. The H level in the most drastically C3H-downregulated transgenic poplar falls well beyond the H:G:S compositional bounds of normal angiosperms. The response observed here, in poplar, differs markedly from that reported for alfalfa where the S:G ratio remained almost constant even at substantial H levels, highlighting the often differing responses among plant species.

## Introduction

**Fig. 1:**
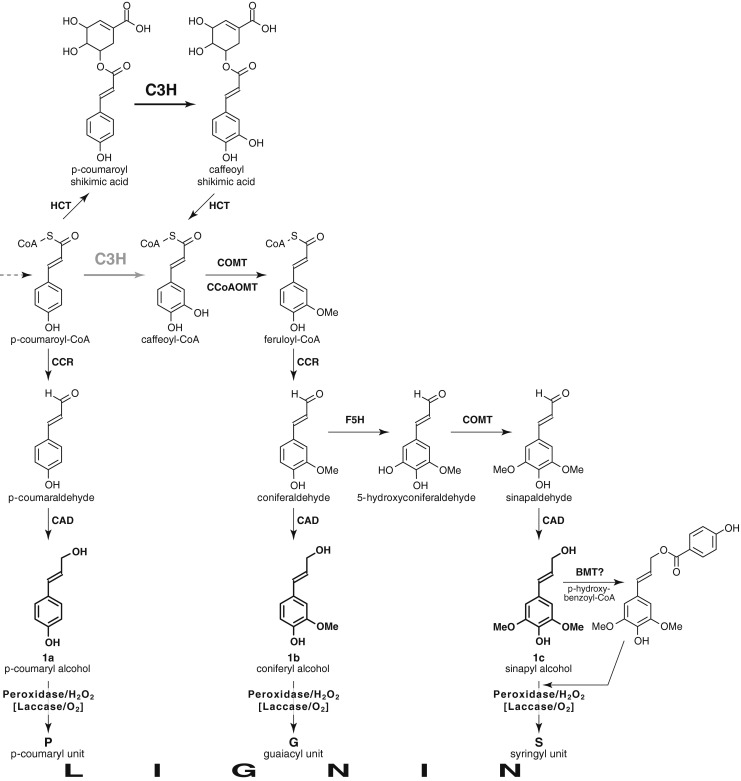
Partial monolignol biosynthetic pathway based on Ref. [4]. The pathway generates the primary monolignols **1** and, following incorporation into lignins via radical coupling reactions, the lignin H, G, and S units. *p*-Coumarate 3-hydroxylase (C3H), the enzyme of major focus here, is now understood to operate on *p*-coumarate esters of shikimic acid (shown), quinic acid, or possibly others, themselves produced by *p*-hydroxycinnamoyl-CoA: quinate/shikimate *p*-hydroxycinnamoyl transferases (HCTs) [11], rather than directly on *p*-coumaroyl-CoA as previously thought (shown in *gray*). Enzymes on the remainder of the pathway have their standard abbreviations [4] except for BMT. *CAD* cinnamyl alcohol dehydrogenase, *CCoAOMT* caffeoyl-CoA *O*-methyltransferase, *CCR* cinnamoyl-CoA reductase, *COMT* caffeic acid *O*-methyltransferase, *F5H* ferulate 5-hydroxylase. *BMT* is the assumed *p*-hydroxybenzoyl-CoA: monolignol transferase, an analog of the recently characterized putative *p*-coumaroyl-CoA: monolignol transferase [50], which acylates a fraction of the monolignols, mainly sinapyl alcohol, in poplar prior to the export of lignin monomers to the wall; incorporation of these *p*-hydroxybenzoate conjugates into lignins results in the *p*-hydroxybenzoylation of the polymer that is readily seen in NMR spectra (Fig. 2)

The impact of perturbing lignification has been studied in several plant species using a variety of genomic strategies [1–10]. One of the early enzymes in the pathway is 4-coumarate 3-hydroxylase (C3H), a crucial hydroxylase that provides entry to the synthesis of guaiacyl (G) and syringyl (S) lignin precursors from the non-methoxylated *p*-hydroxyphenyl (H) branch of the pathway (Fig. 1). Guaiacyl and syringyl subunits dominate the structural composition in most dicot plant species and, in fact, the monolignol *p*-coumaryl alcohol contributes negligibly (typically <1 % of the monomers) to the overall lignin macromolecular polymer but can accumulate significantly in C3H-downregulated plants. C3H is now believed to operate on *p*-coumarate esters of shikimic acid (Fig. 1) or quinic acid (not shown), themselves produced by hydroxycinnamoyl transferases (HCTs), as initially established in *Arabidopsis* [11].

The C3H-deficient *Arabidopsis ref8* mutant [12] was shown to have lignin composed almost solely of *p*-hydroxyphenyl (H) units and no detectable guaiacyl or syringyl components, as determined by degradative methods. These observations are consistent with the ascribed role of the hydroxylase on the pathway, directing the monolignol flux toward coniferyl and sinapyl alcohol formation. However, difficulty in securing sufficient cell wall material from the *Arabidopsis ref8* mutants, which are severely stunted [12, 13], has limited more detailed structural studies (i.e., NMR) of the resultant lignins.

**Fig. 2:**
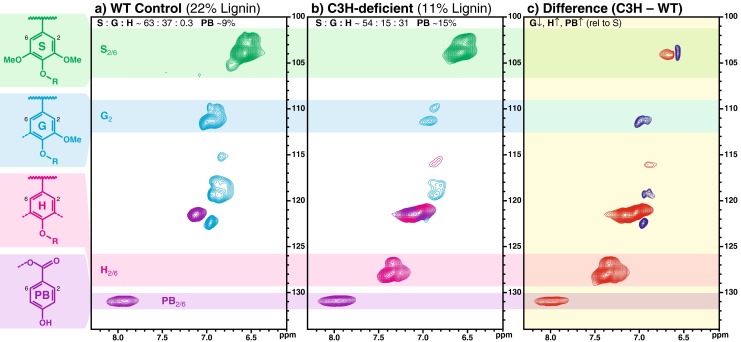
2D NMR spectra revealing lignin unit compositions. Partial short range ^13^ C–^1^ H (HSQC) correlation spectra (aromatic regions only, aromatic units colored as per their structures at the left) of acetylated cellulolytic enzyme lignins (Ac-CELs) isolated from **a** the wild-type control and **b** the most highly C3H-deficient poplar line, along with **c** a 2D difference spectrum, with *red* being elevated and *blue* being depleted components (i.e., not color-coded by structure), nulling the syringyl correlations for ready visualization of the G depression and PB elevation relative to S. Traces of *p*-hydroxyphenyl (H) units are seen in the typically syringyl/guaiacyl (S/G) lignin in the wild-type poplar, whereas H units are seen as significant contributors to the spectrum from the transgenic. *p*-Hydroxybenzoate units (PB), also a feature of poplar lignins, are identified. Semiquantitative volume integrals on the plots are also given in Table 1

In contrast, sufficient plant materials were more readily available from transgenic plants of the forage legume alfalfa (*Medicago sativa*) in which C3H levels had been reduced to as low as 5 % of the wild-type (WT) transcript levels [10]. Interestingly, these substantial depressions in C3H transcript were not accompanied by serious impairment in growth phenotypes, and structural analysis of the lignin revealed striking differences in the lignin composition, despite this normal growth [14]. The major difference was the highly elevated level of H units, up to ∼ 65 % vs. only ∼ 1 % (by NMR) in the WT controls. The altered monomer distribution also resulted in significant changes to the composition and structure of the lignin polymer, as determined by an analysis of the major interunit linkages and non-phenolic endgroups [14]. In short, the perturbations resulted in lower levels of key β-aryl ether units that were augmented by higher levels of phenylcoumarans and resinols. The C3H-deficient alfalfa lignins were also devoid of spirodienones (β–1 coupling products), highlighting the significant differences in the reaction course for *p*-coumaryl alcohol vs. the two normally dominant monolignols, coniferyl and sinapyl alcohols. A larger range of dibenzodioxocin structures (5–5/4–O–β coupled units) was also evident, in conjunction with an approximate doubling of their proportion; two classes, H-H-H and H-H-G dibenzodioxocins, were well resolved from the normal G-G-G analogs. More recently, similar compositional changes were noted in downregulated HCT alfalfa [15].

Although gymnosperms (softwoods) are highly divergent from angiosperms and have no S component to their lignins, their analysis is also instructive. Downregulation of HCT in pines clearly demonstrated elevated levels of H units, up to as high as 23 % (by NMR) [16]. Structural differences in the polymer included an increase in resinols, a reduction in dibenzodioxocins, and the elevated presence of glycerol end-groups. This is interesting given that HCT is directly associated with C3H in the pathway, and its downregulation results in changes that might be expected of the misregulation of C3H, which has not, to date, been the target of downregulation in gymnosperms. Lignins from C3H-downregulated hardwoods have also not previously been structurally characterized, although the development of such transgenics and the physiological and compositional impacts have been described [17, 18].

Structural effects on this key cell wall polymer, lignin, are important to delineate in poplar as, in addition to being excellent model plants for understanding the effects of gene misregulation, poplars and related hardwoods are established feedstocks for pulp, paper, and solid wood industries, and are currently being considered as a primary plantation feedstock for liquid fuel platforms in several regions of the world. Despite its promise, the inherent heterogeneous nature of the supramolecular structures occurring in plant cell wall matrices currently limit poplar’s use in biofuel applications. Much of this is inextricably linked to the innate structural characteristics of the lignin and the modifications that occur to the lignaceous substrate during the requisite pretreatment process (e.g., steam explosion and dilute acid treatment) that is needed to successfully convert wood to liquid fuels. As such, modifications to lignification are strategically driven. As should be evident, extrapolating from model plants, such as *Arabidopsis*, to actual plantation bioenergy crops/trees is not always straightforward. Here we report not only on the common features but also on the striking differences, delineated from structural characterization of lignins in C3H-downregulated poplar, highlighting the need to appreciate the often differing responses between plant species. We also highlight some interesting effects noted in the abrupt onset of H lignin accumulation with decreasing lignin level, which fundamentally addresses the biochemical flux through the pathway and highlights the need to understand the substrate specificities of the individual enzymes on a plant-by-plant basis.

## Materials and Methods

### General

All chemicals were purchased from Aldrich (Milwaukee, WI, USA), unless otherwise noted.

### Cell Wall Analytical Methods

Klason lignin determination (acid-insoluble and acid-soluble), analytical thioacidolysis, and the values presented herein for comparison in Table 1 were as reported previously [17].

**Table 1:** Data for WT control and the most C3H-deficient poplar line

Measure	WT	C3H
Klason lignin^a^		
Acid-insoluble lignin	21.3 %	9.5 %
Acid-soluble lignin	2.5 %	1.0 %
Total lignin	23.8 %	10.5 %
Thioacidolysis^a^		
H (*p*-hydroxybenzyl unit)	0.2 %	20.6 %
G (guaiacyl unit)	35.5 %	19.3 %
S (syringyl unit)	64.3 %	60.0 %
G/S ratio	0.55	0.32
NMR		
H (*p*-hydroxyphenyl unit)	0.3 %	31 %
G (guaiacyl unit)	37 %	15 %
S (syringyl unit)	63 %	54 %
G/S ratio	0.59	0.28
PB (*p*-hydroxybenzoate)^b^	9 %	15 %
**A** (β–O–4)	88.8 %	88.9 %
**B** (β–5)	4.9 %	4.3 %
**C** (β–β)	6.3 %	6.8 %
**X1** (cinnamyl alcohol endgroup)^b^	1.1 %	1.1 %

^a^Ref. [17], C3H is line C3H-14

^b^ *p*-Hydroxybenzoate levels (volume integrals) are expressed as a fraction of H + G + S; X1 is as a fraction of A+B+C

### Plant Materials

Transgenic poplar (*Populus alba* × *grandidentata*) trees perturbed in C3H transcript abundance and their corresponding enzyme activity were generated as described previously [17]. Specifically, the C3H-deficient line evaluated herein is line C3H-14 in that reference.

### Lignin Isolation

Stems were harvested from control (WT) and the most heavily C3H-deficient poplar lines following one complete year of greenhouse growth. Lignins were isolated using methods largely described previously [19]. Briefly, poplar stem wood was separated from the pith material, roughly cut, and then 12.144 and 8.783 g (WT and C3H, respectively) were ground in a SPEX freezer mill (2 min, impact setting 10) to give 11.935 and 8.454 g of wood meal. The majority of this material (7.158 and 8.532 g) was sequentially extracted with acetone and then methanol for 4 h using a Foss (Eden Prairie, MN 55344) Soxtec system 2045, yielding 6.483 and 7.664 g of extractive-free wood meal (cell walls). Each of these were then ball-milled for 2.5 h (in 0.5 h on/0.5 h off cycles to avoid excessive sample heating) using a custom-made ball mill using an offset 1/4 HP Dayton motor running at 1,725 rpm with rotating (0.2 Hz) stainless steel vessels (12.2 cm diameter, 11.4 cm high) containing ∼ 3.7 kg 5-mm stainless steel ball bearings; total weight of the jar and bearings is ∼ 6.15 kg. The ball-milled walls (6.00 g each) were then digested at 30°C with crude cellulases (Cellulysin, Calbiochem, San Diego, CA, activity 11,000 units/g, lot #B29887, 30 mg/g of sample, in pH 5.0 50 mM acetate buffer, 50 ml in three tubes each, 3 × 48 h, fresh buffer and enzyme each time) resulting in a ‘cellulolytic enzyme lignin’ (CEL) fraction [20] containing all of the lignin and residual polysaccharides totaling 1.5618 g (26.7 % of the original cell wall, WT) and 0.822 g (12.0 %, C3H) (Table 1). These CELs (101 and 100 mg) were acetylated by first solubilizing the walls at 22°C overnight in DMSO (2 ml) and N-methylimidazole (1 ml) with stirring and then adding Ac_2_O (0.5 ml) with stirring for 1.5 h at room temperature. The resulting brown-colored solutions were dropped onto water (380 ml) with stirring to precipitate the acetylated CELs. The resulting suspensions were centrifuged at 7,000 rpm for 15 min. The pellets were collected and washed with water two times and then the water was removed by lyophilization to yield 105 (WT) and 69.4 mg (C3H) of Ac-CELs.

### NMR Spectroscopy

The NMR spectra were acquired on a Bruker Biospin (Rheinstetten, Germany) DMX-750 instrument fitted with a sensitive cryogenically-cooled 5-mm TXI ^1^ H/^13^ C/^15^ N gradient probe with inverse geometry (proton coils closest to the sample). Lignin preparations (Ac-CELs, 40 mg) were dissolved in 0.5-ml CDCl_3_; the central chloroform solvent peak was used as internal reference (*δ*_C_ 77.0, *δ*_H_ 7.26 ppm). We used the standard Bruker implementations of the traditional suite of 1D and 2D (gradient-selected, ^1^ H-detected) NMR experiments for structural elucidation. HSQC experiments at 750 MHz used Bruker’s ‘hsqcetgpsisp.2’ pulse program with adiabatic pulses to minimize the sensitivity to ^1^J_C–H_ variations and produce uniform excitation across the spectral range. Spectra had the following parameters: acquired from 11 to 1 ppm in F_2_ (^1^ H) using 2,696 data points (acquisition time 150 ms), 200 to −20 ppm in F_1_ (^13^ C) using 512 increments (F_1_ “acquisition time” 6.2 ms) of 44 (WT) or 104 (C3H) scans with a 1-s interscan delay, total acquisition time of 7 h 21 min (WT), or 17.5 h; the *d*_24_ delay was set to 0.86 ms (∼1/8 *J*, *J* = 145 Hz). Processing to a final data size of 2k × 1k used typical matched Gaussian apodization (LB = −0.15, GB = 0.001) in F_2_ and cosine-bell apodization in F_1_.

**Fig. 3:**
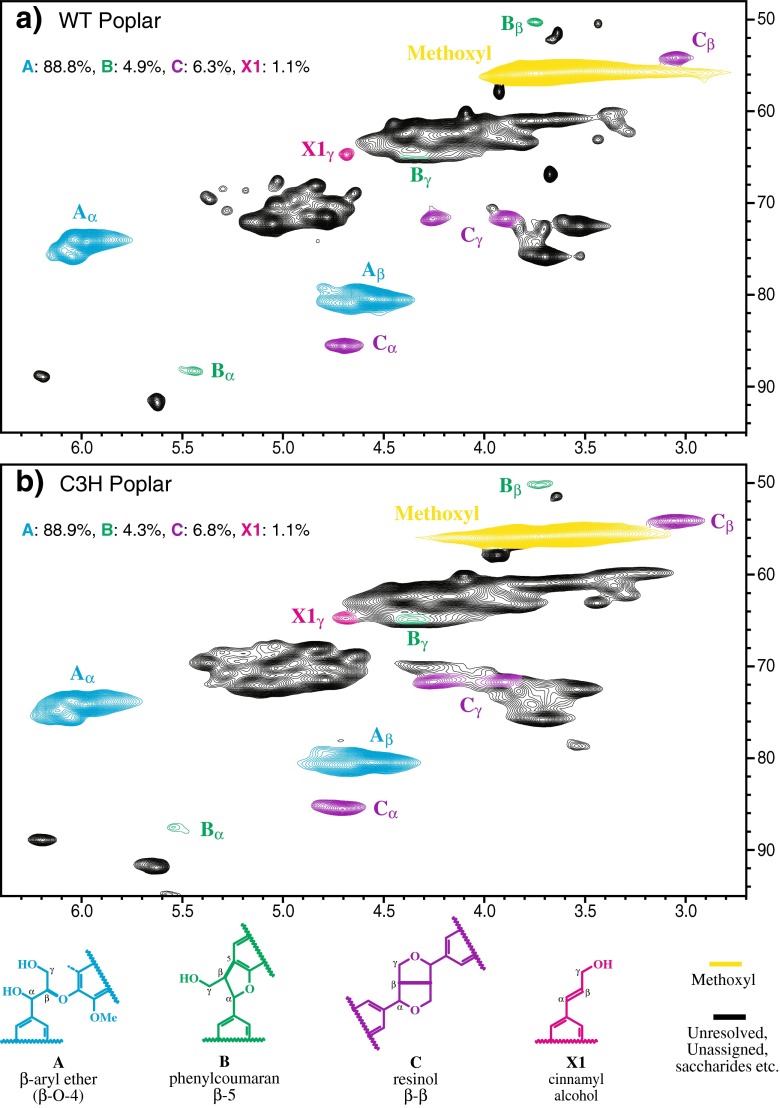
2D NMR spectra revealing lignin interunit distributions. Partial short range ^13^ C–^1^ H (HSQC) spectra (side-chain regions) of acetylated cellulolytic enzyme lignins (Ac-CELs) isolated from **a** the most highly C3H-deficient line and **b** the wild-type control, showing similar distribution of interunit linkages. Spectral contours are color-coded according to the structures below. Percentages of the major units **A**–**C**, **X1**, are from volume integrals and are uncorrected

Volume integration of contours in the HSQC plots was accomplished using Bruker's TopSpin 3.1 (Macintosh) software, essentially as described previously [14, 21]. For quantification of H:G:S ratios (Fig. 2), only the carbon-2 correlations from guaiacyl (G) units and the carbon-2/6 correlations from syringyl (S) or *p*-hydroxyphenyl (H) units were employed, and the guaiacyl integrals were logically doubled. No correction factors were deemed necessary after noting only slight deviations from 1:1:1 volume integral ratios in a range of model dimers and trimers with mixed H/G/S units. For quantification of the various interunit linkage types, the following well-resolved contours (see Fig. 3) were integrated: **A**α, **B**α, **C**α, and **X1**γ. Integral correction factors were not used (as currently, there is no reliable way to determine ‘response factors’)—it is only the volume integrals that are reported; relative compositional changes are thus accurate, but absolute quantification is not possible, primarily because there are no absolute methods to allow validation or the calculation of ‘correction factors’.

## Results and Discussion

The data reveal both major compositional shifts and subtle interunit linkage differences between the syringyl–guaiacyl lignins in normal WT poplar vs. the *p*-hydroxyphenyl-elevated lignins in the most highly downregulated C3H-RNAi transgenic. Such structural differences provide some fundamental basis for explaining differences in digestibility and bioenergy performance of C3H-deficient plants. Most of the structural analysis comes from 2D NMR spectra of the acetylated ‘cellulolytic enzyme lignins’ (CELs) derived by treating the (ball-milled) whole wall material with crude cellulases to remove a large fraction of the polysaccharides while retaining essentially all of the lignin (see “Materials and Methods”).

### Lignin Levels and Aromatic Unit (H:G:S) Distribution

**Fig. 4:**
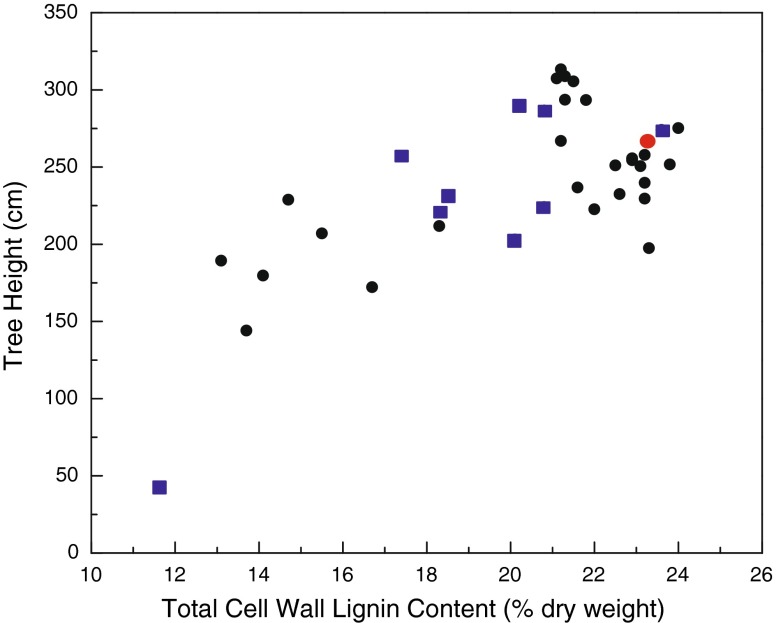
Scatter plot relating poplar height growth to total cell wall lignin content in 8-month-old C3H RNAi-suppressed transgenic and wild-type (*red circle*) greenhouse-grown hybrid poplar trees. Each point represents an average of ten clonally replicated trees from independent transformation events. *Blue boxes* represent transgenic lines previously characterized [17, 51]

**Fig. 5:**
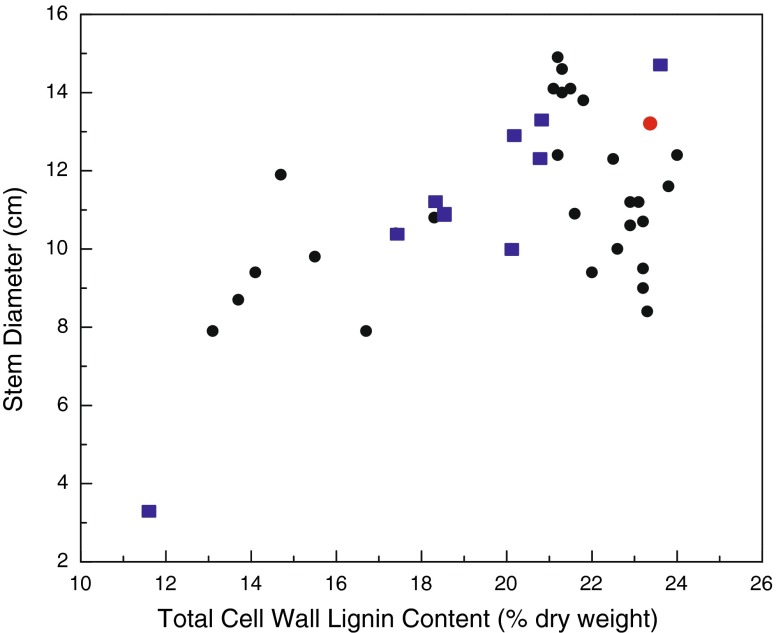
Scatter plot relating poplar diameter growth to total cell wall lignin content in 8-month-old grown C3H RNAi-suppressed transgenic and wild-type (*red circle*) greenhouse-grown hybrid poplar trees. Each point represents an average of ten clonally replicated trees from independent transformation events. *Blue boxes* represent transgenic lines previously characterized [17, 51]

Downregulation of the target *C3H* gene (*C3H-3*) in poplar resulted in the establishment of several transgenic lines, ranging in *C3H* transcript abundance suppression from those near WT levels to as low as 5 % of that in the WT. In most cases, lignification was affected, and in the most severely depressed transgenic lines, the total cell wall lignin contents were ∼ 10 % vs. 22.5 % in the WT control lines (Table 1), as measured by the Klason lignin [17]. In poplar, unlike in other species, the only other gene that has been misregulated and resulted in such a severe reduction in lignin amount is *4CL* [22], which is a key gene that lies upstream of *C3H*. *4CL* suppression resulted in as much as a 45 % reduction in total cell wall lignin and reportedly no impairment in growth [22]. In contrast, reductions in *C3H*, and consequently lignification, resulted in varying effects on growth properties (Figs. 4 and 5) [18]. Although the extreme cases were stunted in growth, it is apparent that it is possible to select genetically modified lines with reduced lignification that grow equally as well as the WT plants. In general, it appears that the tradeoff between growth impairment and lignin content is when lignification is below 17 % total biomass.

Lignification has been shown to begin in the cell corners and middle lamella, with H-rich lignins being deposited early in those regions [23]. Despite this, and although *p*-coumaryl alcohol is regarded as a ‘traditional monolignol’ for lignin biosynthesis, the fact is that dicot ‘bulk’ lignins are always characterized as having extremely low levels of this component [24, 25], typically <1 % (and only 0.3 % as determined by NMR in the WT poplar here, Table 1 and Fig. 2). Downregulation of poplar C3H-3, which provides a key entry into the production of the major, methoxylated, monolignols, therefore appears to limit the overall availability of monomers required for lignification.

More importantly and as anticipated from *a priori* knowledge of the monolignol biosynthetic pathway (Fig. 1) and from the modest number of studies in other plant models (*Arabidopsis*, alfalfa, and pine), C3H downregulation successfully resulted in a relative depletion in the levels of units derived from methoxylated lignin monomers (coniferyl and sinapyl alcohols), and a striking elevation of the levels of *p*-hydroxyphenyl (H) units from the non-methoxylated monolignol, *p*-coumaryl alcohol. Indeed, analysis of the aromatic region of 2D ^13^ C–^1^ H correlation (HSQC) NMR spectra (Fig. 2) showed that the H lignin levels rose to an impressive 31 % of the total lignin units (H + G + S = 100 %), representing an elevation of some two orders of magnitude over the WT level. Interestingly, the elevation in H units was largely at the expense of guaiacyl units, which became the minor component of the three traditional monomer subunits. The difference spectrum in Fig. 2c clearly shows the elevation of the *p*-hydroxyphenyl units along with relative depletion of G units compared to the (nulled) S unit level. In the WT poplar material, the G/S level was 0.59, vs. only 0.28 in the C3H-downregulated transgenic lines. These values compare favorably with relative G/S ratios determined by analytical thioacidolysis (0.55 vs. 0.32) (Table 1) [17], even though the two measurements measure G/S on entirely different fractions of the lignin—the NMR value representing the entire cell wall lignin and the thioacidolysis value representing the G/S of only the monomers released by cleaving β-ether bonds in the lignin. This behavior contrasts strikingly with that observed in alfalfa C3H-downregulated lines where the thioacidolysis G/S stayed essentially constant, going from 2.1 in the control line to 2.0 in both a line with some 55 % H lignin and in a 28 % H-line [14].

### Impact of H:G:S Composition on Lignin Structure

The side-chain region only peripherally reflects the changes in the H:G:S distribution, but is rich in details regarding the types and distribution of interunit bonding patterns present in the lignin fraction. The control poplar lignin spectrum (Fig. 3a) is typical of a guaiacyl–syringyl lignin containing residual polysaccharides [26]. The HSQC spectrum resolves most of the correlations for the various interunit linkage types in the polymer, the exception being the complex γ region where only the correlations from the phenylcoumarans **B** and the cinnamyl alcohol endgroups **X1** are partially resolved. The WT lignin is seen as being rich in β-aryl ether units **A**, with more modest amounts of phenylcoumaran **B** and resinol **C** units, as is typical for all lignins. Spirodienone structures, β–1 coupled units only recently authenticated in lignin spectra [27, 28], were not readily seen in these poplar spectra. Dibenzodioxocin structures resulting from radical coupling of a monolignol with a 5–5 coupled end-unit [29] are also not significant. Finally, the cinnamyl alcohol endgroups **X1**, like the resinols **C**, arise from monomer–monomer coupling, as is usually seen in G/S lignins. Resinols are relatively prevalent; the deceptively strong **X1** γ-C/H correlation peak is due to the sharpness caused by the relative invariance of proton and carbon chemical shifts in such structures where the bonding is on the aromatic ring, well distant from the γ position. We also remain suspicious that polysaccharide correlations can overlap and inflate this integral [21]. For comparisons, we integrate all of these assigned interunit peaks, but sum only those internal chain structures (**A**–**C**) to 100 %; **X1** levels are reported relative to that sum.

In contrast, the C3H-deficient lignin has a spectrum that is only subtly different, but these differences are consistent with the observed compositional shifts. The relative intensity differences (seen more easily from the volume integral data in Table 1) signify only minor structural changes. The major difference is that the phenylcoumaran **B** appears (Fig. 3) to be quite depleted. This may seem realistic given the observation above that G levels (relative to S levels) are reduced compared to WT—phenylcoumarans are derived from coupling of a monolignol with a G units. However, closer inspection and integration of the region shows that the difference is only minor (4.3 vs. 4.9 % **B**), and the **B**α correlation has ‘smeared out’. Logically this is because the H units, which were responsible for most of the G depletion, can also form phenylcoumarans **B**, but their C/H chemical shifts differ subtly, as are more readily seen by the long (in the ^1^ H dimension) correlation peak for **B**β in the C3H-deficient transgenic vs. the WT (Fig. 3). Somewhat surprisingly, we did not see any obvious elevation in the barely detectable dibenzodioxocin levels (not shown). Thus, even the massive compositional changes observed here, when G lignin is displaced by H subunits in these poplars, do not greatly impact the distribution of interunit linkages in the polymer. Far more substantial changes are encountered when the H unit elevation is also at the expense of S units, as in the case of alfalfa [14].

Analyses of the data presented here, along with those published previously on these transgenics [17], highlight another intriguing phenomenon. Reduction in C3H transcript levels did not alter the S:G, nor result in any significant buildup in H lignin, except in the most extremely repressed line (with ∼ 5 % of the C3H transcript levels found in the corresponding control trees), at which point the H lignin content soared. However, the lignin level steadily declined with the suppression in C3H level. C3H is, therefore, a limiting enzyme, governing the flux though the pathway to the ultimate monolignols. However, the flux still shunts through to the normal G/S pathway in poplar until, presumably, H precursors build up to such a point that they are directed through the reductive pathway to *p*-coumaryl alcohol which is exported to the wall for lignification. The control mechanisms are unknown. It is also not obvious why the H level, unlike in alfalfa [14], is largely at the expense of G units in poplar, and not S units. There is no way to know if there is a similar threshold phenomenon occurring in *Arabidopsis*, as downregulated transgenics have not been created; the C3H-deficient *ref-8* knockout mutant obviously cannot direct flux through the G/S branches of the pathway so the only monolignol available for lignification is *p*-coumaryl alcohol [12]. It is also not clear if the poplar observation differs from that in alfalfa, although it appears to. Thus, at both modest and high C3H downregulation levels, H units are strikingly increased (with the level corresponding to the C3H level) in alfalfa [14], and even modest downregulation of HCT appears to result in higher H lignin levels [15]. Again, a difference is that the S/G remains essentially constant. Regardless, plant-specific effects are evident. Such effects are reminiscent of observations with the upregulation of ferulate 5-hydroxylase (F5H) in *Arabidopsis* vs. poplar. In poplar, syringyl levels as high as ∼ 97.4 % are achieved [30], but there is no evidence of products of incomplete methylation, i.e., no benzodioxanes resulting from accumulation and incorporation of 5-hydroxyconiferyl alcohol into the lignin in these transgenics. In contrast, in *Arabidopsis*, upregulation of F5H to produce lignins with equivalently low G levels (∼3 %) [31], results in lignins with a considerable component (10 %) derived from 5-hydroxyconiferyl alcohol [32]. In other words, at these levels of F5H upregulation, the poplar COMT can apparently keep pace with the increased flux of 5-hydroxyconiferaldehyde, whereas in *Arabidopsis* it cannot. Again, this highlights the need to evaluate individual systems independently, as model systems are just that, models from which fundamental information is gained, but which should not be perceived as supplying universal principles.

### Impact on *p*-Hydroxybenzoate Esters

An extremely important feature of poplar is that, unlike other dicots and in fact most other hardwoods except those species with the genera *Salix*, *Populus*, or *Palmae* (although palms are actually monocots), it has *p*-hydroxybenzoates acylating a fraction of its lignin side-chain [21, 33–39]. Like their *p*-coumarate counterparts in grasses [21, 40–43], *p*-hydroxybenzoates are almost entirely free phenolic pendant units on lignin and are exclusively attached to lignin side-chains at the γ-positions. As with acetates and *p*-coumarates, they arise on lignins from lignification with preformed monolignol *p*-hydroxybenzoate conjugates that must be exported to the cell wall along with the traditional (unacylated) monolignols [21, 44, 45]. We have shown, in largely unpublished work, that these *p*-hydroxybenzoates are almost exclusively on syringyl units in the above natural plants. Obviously, the effects of C3H downregulation on such *p*-hydroxybenzoates have not been addressed previously, as they do not appear on either *Arabidopsis* or alfalfa lignins. Elevated *p*-hydroxybenzoate levels were, however, previously observed in CCoAOMT-downregulated poplars, which had slightly lower lignin contents and were additionally slightly increased in S units [37].

Given the lignin content reduction, and also the skewing of the lignin monomer distribution away from largely coniferyl alcohol to *p*-coumaryl alcohol, the effect on *p*-hydroxybenzoates was not readily predictable. In fact, as detailed in Table 1 and Fig. 2, the levels (relative to total lignin H + G + S units) were substantially elevated in the C3H-downregulated transgenic (∼15 %) vs. the WT control (∼9 %), as measured by volume integration of the PB2/6 correlations. The difference spectrum in Fig. 2c also clearly shows the elevation of the *p*-hydroxybenzoate (along with relative depletion of G units) compared to the (nulled) S unit level. It remains to be determined whether *p*-hydroxybenzoates continue acylating solely S units in these transgenics. Also, although *p*-hydroxybenzoates, like their *p*-coumarate analogs, are prone to radical transfer reactions rather than radical coupling in the radical-limited environment of lignification [46–48], and therefore do not etherify into the lignin polymer by cross-coupling with G or S units, it might be anticipated that they are more compatible with cross-coupling with H units. However, no evidence of this can be found—the chemical shifts here are totally compatible with the phenolic-OH of such units having been entirely acetylated (and therefore originally free phenolic). Either they are not, in fact, compatible with radical coupling reactions with H units, or there are sufficient G and S units persisting in the polymer during lignification that radical transfer remains the primary mechanism. Unless almost complete downregulation of C3H can be achieved to produce lignins with H levels substantially higher, as in the C3H mutants in *Arabidopsis* [49] or in the most highly downregulated alfalfa lines [14], the compatibility of *p*-hydroxybenzoates with cross-coupling to H units, *in planta*, is unlikely to be determined. It would also be of interest to determine whether, in the absence of sinapyl alcohol, *p*-hydroxybenzoates would acylate *p*-coumaryl alcohol via a presumed transferase. We have recently demonstrated that the *p*-coumaroyl-CoA: monolignol transferase implicated in grasses has a substrate preference for *p*-coumaryl alcohol [50], even though *p*-coumarates in grasses have not been observed on the (minor) H units, and actually appear on S and G units in an ∼ 90:10 ratio in corn [42, 43], for example. The presumed analogous *p*-hydroxybenzoyl-CoA: monolignol transferase in poplar has not yet been identified but warrants further examination.

### Implications

Lignins elevated in *p*-hydroxyphenyl (H) units are produced by C3H downregulation in poplar, in plants that also have substantially lower lignin contents. Most of the relative H unit elevation is at the expense of G units rather than S units, quite unlike the case in C3H-downregulated alfalfa in which the G/S ratio changed only slightly. NMR analysis of the lignins suggests that *p*-coumaryl alcohol undergoes coupling and cross-coupling reactions that are, for the most part, analogous to those of the normally dominant monolignols, coniferyl and sinapyl alcohols, in these H/S-rich, G-depleted lignins. *p*-Hydroxybenzoates, pendant units acylating lignin side-chains, are also markedly elevated on a lignin basis. However, similar findings were not apparent in transgenic lines where perturbation in lignification was not as significant, which is particularly intriguing. This suggests that general conclusions about flux through the monolignol pathway cannot be assumed based on findings in other species or unique plants. This conclusion is substantiated by findings demonstrating considerable differences between the responses to C3H downregulation in poplar vs. *Arabidopsis* and alfalfa, and highlights the importance of delineating the effects directly in the plant species of interest along with evaluating the impact of the lignin changes on various conversion processes. Moreover, the results show that opportunities exist to select lines with partial repression of lignification without deleterious impacts on growth phenotypes and that such lines can have positive implications for bioenergy applications [51]. It is equally apparent that not all genes repress lignification similarly or have common consequences on growth.
